# Minor role of BRCA2 mutation (Exon2 and Exon11) in patients with early-onset breast cancer amongst Iranian Azeri-Turkish women

**Published:** 2014-02

**Authors:** Nahid Karimian Fathi, Mahmood Shekari Khaniani, Vahid Montazeri, Sima Mansoori Derakhshan

**Affiliations:** 1 Medical Genetic Department Medical Faculty, Tabriz University of Medical Sciences, Tabriz, Iran; 2 General Surgery Department Medical Faculty, Tabriz University of Medical Sciences, Tabriz, Iran

**Keywords:** *BRCA2*, Breast cancer, Iranian population, Mutation detection, Sequencing, SSCP

## Abstract

***Objective(s): ***Breast cancer is the most common cancer in women. Every year, one million new cases are reported worldwide, representing 18% of the total number of cancer in women. Hereditary BRCA1 and BRCA2 mutations account for about 60% of inherited breast cancer and are the only known causes of hereditary breast cancer syndrome. The aim of this study was to determine the frequency of BRCA2 (exon2 and exon11) gene mutation in patients with early-onset breast cancer among Iranian Azeri-Turkish women.

***Materials and Methods: ***We obtained clinical information, family history and peripheral blood from 110 women under the age of 45 with early-onset breast cancer for scanning germline mutations in the exon2 and 11 of BRCA2 genes which comprises over 50% of the gene. Single-strand conformation polymorphism assay was used in order for screening potential mutations on amplified regions followed by direct sequencing analysis to determine the genotypes.

***Results: ***Overall, 11 sequence variants were identified in this study group, including four homozygotes and seven heterozygotes silent substitution of c.3807T to C, p.Val1269Val (rs543304).

***Conclusion: ***Mutations in BRCA2 were surprisingly infrequent in the early onset breast cancer patients among Iranian Azeri-Turkish women.

## Introduction

Breast cancer is the most prevalent malignancy and primary cause of death due to cancer in women (1). Some studies in Iran suggest that breast cancer affects Iranian women in at least one decade younger than the women in developed countries (2). Genetic susceptibility to cancer is due to inactivation of germline mutations in tumor suppressor and DNA repair genes, which result in uncontrolled cell division. About 5–10% of breast and ovarian cancers occur as a result of highly penetrant germline mutations in these genes (3). Two major breast cancer susceptibility genes are BRCA1 and BRCA2, located on chromosomes 17 and 13, respectively and both perform function as tumor suppressor genes. They are involved in the control of double-strand break repair and homologous recombination in response to DNA damage (4). Women with mutations in BRCA1 or BRCA2 gene are 3 to 7 times more likely to develop breast cancer than the women without mutations in those genes, with excessive relative risks of about 30-fold for early onset diseases (before the age of 45) (5). Carriers of BRCA1 and BRCA2 mutations are also at an increased risk of other cancers such as ovarian cancer (1). Germline mutations in these genes usually result in truncation or absence of the protein that increases up to 90% lifetime risk of breast cancer in the carrier women. Studies show that BRCA2 mutations are generally less common than that of BRCA1    (6). In general, the identification of the breast cancer susceptibility genesand genetic testing for mutations in both genes are the basis for estimating the disease risks for women with a strong family history of breast cancer and will provide important information for the prevention and treatment of familial breast cancer (7). Currently, over 900 different alterations have been identified in BRCA2 gene (8). Mutations in this gene have been reported in various populations, some of which being unique to each population (9). 

**Table 1:** Sequences of the utilized primers and Tm, PCR product sizes

No	Primer names	Primer sequences	Tm	PCR.P
1	Exon2-Fwd	*5'-AATGCATCCCTGTGTAAGTGC -3'*	54	161
2	Exon2-Rev	*5'-TCAATACCTGCTTTGTTGCAG -3'*		
3	Exon11A-Fwd	*5'-ATGGTTTTATATGCAGACACAGG -3'*	55	159
4	Exon11A-Rev	*5'-TATATTCAAGGAGATGTCCGATTT -3'*		
5	Exon11B-Fwd	*5'- GGTGCTGTGAAACTGTTTAGTGATA -3'*	58.7	177
6	Exon11B-Rev	*5'- TCGGTTGTAATATCAGTTGGCATTTATTAT -3'*		
7	Exon11C-Fwd	*5'-GACAAAAATCATCTCTCCGAAAA -3'*	56	199
8	Exon11C-Rev	*5'- CATTGGATATTACTTTGGAAAAACTAG -3'*		
9	Exon11D-Fwd	*5'-CATAACCAAAATATGTCTGGATTGGAG -3'*	57.9	186
10	Exon11D-Rev	*5'-TAATGAAGCATCTGATACCTGGAC -3'*		
11	Exon11E-Fwd	*5'-GGGAGTGTTAGAGGAATTTGATTT -3'*	54	269
12	Exon11E-Rev	*5'-TTCTGAAGAACCACCTTCAACA -3'*		

The cost and time required for mutation analysis are reduced considerably when founder mutations are identified for a specific ethnic group. The aim of this study was to determine the frequency of mutation in exon2 (the first translated exon of BRCA2 gene that have not previously been studied in Iran) and exon11 (which comprises over 50% of the gene) of BRCA2 gene in patients with early-onset breast cancer in Iranian Azeri-Turkish women.

## Materials and Methods

***Acquisition of Clinical Information***

Corresponding clinical information about each proband and her family was obtained by a questionnaire and according to BRCA2 inheritance matter, pedigrees were designed. The questionnaire allowed specifying the family history, sex, the age of being diagnosed with breast cancer and bilateral or unilateral status. In addition, the questionnaire distinguished between ductal carcinoma *in situ* and invasive breast cancer in the proband. 

***Case selection***

Overall 110 patients with diagnosed breast cancer under the age of 45 were studied. Breast cancer cases with Iranian Azeri-Turkish ethnicity aged below 45, without the history of radiotherapy or smoking were included in this study. Only breast cancer pateints with primary breast tumor were included (Patients with their breast tumor mass being metastasis from other primary cancers were excluded from the study).

Twenty-one women with a familial history of breast cancer in their immediate families, and 89 women with sporadic breast cancer being referred from the department of Cancer Surgery Noor Nejat Hospital in Tabriz, Iran during 2011–2012 were selected for the study. 

Peripheral blood samples (2 ml) from all patients were obtained in EDTA coated vials after giving the informed consents that was approved by the Ethical Committee of the Tabriz University of Medical Sciences.

***DNA isolation and mutation analysis***

Genomic DNA was extracted from peripheral blood lymphocytes by the salting-out method  (10). Six sets of primers were designed to screen the exon2 and 11 of BRCA2 gene. The primers for amplifiction of exon2 produce a PCR product with 161bp. Primers for exon11 of BRCA2 were designed by hot point selection according to number of the times. A specific mutation has been recorded in BIC (Breast Cancer Information Core) database and their sequences and the size of PCR products are depicted as Table 1. We analyzed BRCA2 exons2 and 11 by single-strand conformation polymorphism (SSCP) assay after amplification of genomic DNA with forward and reverse primers of exon2, exon11A, exon11B, exon11C, exon11D and exon11E. PCRs were performed with genomic DNA containing 50 ng of extracted DNA, 0.5 l of both forward and reverse primers at 10 pmol/l, 5 l of red master mix (AmpliconIII Cat#180301), and distilled water being added to a final volume of 10 l. In order for amplification, each sample was denatured for 5 min at 95°C, followed by 32 cycles of denaturation at 95°C for 30 sec, annealing (at 57°C for primers 11A٫ 11C and 11D; at 58.4 °C for primer 11B; and at 59°C for primer 11E and primer 2) for 30 sec, with extension at 72°C for 30 sec, and a final extension step of 5min at 72°C on an Bio-Rad thermal cycler (My Cycler^TM^ thermal cycler, USA). The PCR products were subsequently resolved by electrophoresis on 1% agarose gel and visualized using ethidium bromide.

**Figure 1:**
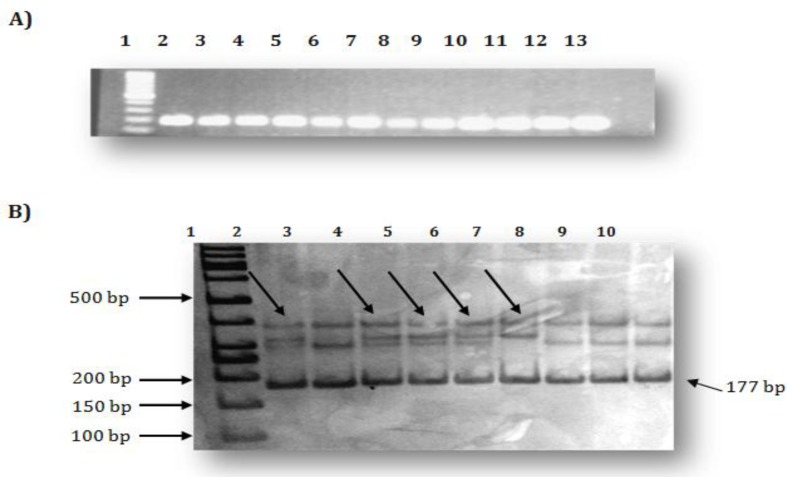
SSCP analysis of exon11B of the BRCA2 gene: A. PCR products of exon11B of the BRCA2 gene (lane 1: 100bp DNA ladder; lanes 2-13: patients PCR products); B. SSCP profiles (Lane 1: 50bp DNA ladder; lanes 2, 4, 5, 6 and 7: SSCP variant; lanes 3, 8, 9 and 10: normal SSCP pattern)

**Figure 2:**
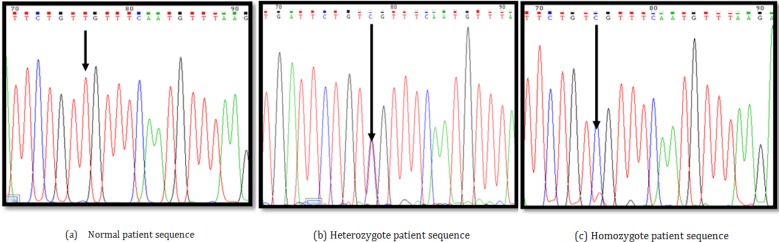
Sequencing analysis of SSCP variants. Shows DNA sequencing from positive PCR-SSCP samples (a. Normal patient sequence, b. Heterozygote patient sequence, and c. Homozygote patient sequence)

For the SSCP analysis, samples were diluted (1:1) in formamide dye (98% formamide, 10 mM NaOH, 0.05% bromophenol blue and 0.05% xylene cyanol). Eight μl of each sample was denatured for 10 min at 95°C, cooled rapidly on ice and separated on a non-denaturing 12% polyacrylamide gel [20 × 20 cm; containing 15ml acrylamide 40% (38.5-1.5 acrylamide: bisacrylamide), 10× TBE, 3% glycerol, 600 μl 10%APS, 40 μl TEMED], and run at 10-12.5 volts/cm for 16–20 hrs in 1x TBE at 4^o^C. The gel was subjected to silver staining in order to visualize the SSCP band patterns (Figure 1).

## Results

One hundred ten patients with early onset breast cancer (<45 age) were recruited for the study. The patient's ages ranged from 27 to 45 years with a mean age of 37±4 years. Out of 110 cases, 17% was familial breast or ovarian cancer and the remaining had not any related person diagnosed with breast or ovarian cancer.Except one case (0.9%)all of the patients had unilateral breast cancer. 

In total, 11 sequence variants were identified in the study group, including 4 homozygotes and 7 heterozygotes of c.3807T to C, p.Val1269Val (rs543304) silent substitution (Figure2), being previously identified in other populations but not in North West of Iran and were reported to the BIC and NCBI databases. No frame shift or missense mutations were detected in any of the exons 2 and 11 of BRCA2 gene in early onset breast cancer patients in Iranian Azeri-Turkish women.

## Discussion

Differences in the incidence and the mortality of cancer result from the differences in genetics and epidemiologic risk factors. Germline mutations in BRCA1 and BRCA2 have been identified among many races and ethnic groups and the frequency of mutations varies between these groups. Some of the differences in cancer risks between the populations may be the result of founder mutations in such genes. when the founder mutations are identified in a specific ethnic group, the cost and time required for mutation analysis are reduced considerably (11). Some of these founder mutations can be observed in Ashkenazi Jewish breast cancer patients and it has been suggested that mutations in the BRCA genes among the Iranians might be different from that of other populations (12).

Few studies  (2, 8, 9) are available describing the spectrum of BRCA2 mutations among Iranian populations; hence, this is the first report to portray the mutations in the BRCA2 gene in the Northwest of Iran. In this study we investigated the mutations in exon2 that is the first translated exon of BRCA2 gene, as well as exon11 which comprises over 50% of the BRCA2 gene in patients with early-onset breast cancer in Northwest of Iran. Screening for the mutations carried out in several groups suggested significant variation of the relative contribution of BRCA1 and BRCA2 genes to hereditary cancer among the populations. Our mutation detection showed one polymorphism (rs543304) in BRCA2 exon11 sequences being screened by SSCP followed by sequencing. 

## Conclusion

This finding indicates the minor importance of mutations in exons2 and 11 of BRCA2 gene (encompassing half of the coding region) in predisposition to breast cancer in Iranian Azeri-Turkish population. Results of our investigation and data had been previously published from Iran indicate that the common BRCA2 mutations are infrequent in Iranian breast cancer patients. 
